# Orum & Armstrong's Teeth

**Published:** 1860-10

**Authors:** 


*Orum & Armstrong's Teeth.*—We received, some time since, a small card of the above manufacturers' teeth. A notice of them should have appeared in last number, but the unfortunate illness of the senior editor prevented.

These specimens are made to imitate the polished surfaces of the English manufacture, to a certain extent, and we judge there has been quite an improvement in this particular, as the polish has not been carried so high as to destroy the appearance of natural structure, so that these teeth present a much closer resemblance to the natural organs. The foreign make, as generally required to match natural teeth, is, we have found, peculiarly deficient in this particular, giving, in most cases, the unmistakable evidences of artificial origin.

The present sample is a decided improvement in these particulars, with a carefully regulated amount of polish, the gum alone being still a little too glassy. The size and form of the crowns are beautifully varied within *practical* limits, for, however correctly formed a tooth may be, it is entirely useless if too large or too small. The sizes before us are extremely well managed, and are those generally required. The lateral joints are quite as accurate as is generally found; but there is room for improvement in this particular in all artificial teeth with gums, or else conundrum wheels must be made of better crystal than we have had the good fortune to procure.

We have never seen better single gum plate teeth than those now made by Messrs. Orum & Armstrong. The practice of the present day demands blocks of teeth in sections of various arches, as suitable for plastic work, and we trust these manufacturers have as successfully supplied this want, as they certainly have with the single plate. B.

